# Recreational Vehicle Water Tanks as a Possible Source for *Legionella* Infections

**DOI:** 10.1155/2013/286347

**Published:** 2013-11-26

**Authors:** Christine M. Litwin, Bankole Asebiomo, Katherine Wilson, Michael Hafez, Valerie Stevens, Carl B. Fliermans, Barry S. Fields, John F. Fisher

**Affiliations:** ^1^Department of Pathology, Medical College of Georgia, Georgia Regents University, 1120 15th Street, Augusta, GA 30912, USA; ^2^Department of Medicine, Medical College of Georgia, Georgia Regents University, 1120 15th Street, Augusta, GA 30912, USA; ^3^Respiratory Diseases Branch, Division of Bacterial and Mycotic Diseases, National Center for Infectious Disease, Centers for Disease Control and Prevention, Public Health Service, United States Department of Health and Human Services, 1600 Clifton Rd, Atlanta, GA 30333, USA; ^4^Savannah River Site, Aiken, SC 29802, USA

## Abstract

We investigated recreational vehicle (RV) water reservoirs in response to a case of pneumonia in which *Legionella pneumophila* was cultured both from the patient and a RV reservoir in which he travelled. Water samples processed and cultured at the CDC according to standard protocol were positive for *Legionella* spp. in 4/17 (24%) faucets, 1/11 (9%) water tanks from 4/20 (20%) RVs from three different campsites. *Legionella* spp. that were isolated included *L. pneumophila* (serogroups 1 and 6), *L. anisa, L. feeleii*, and *L. quateriensis*. Environmental controls from the potable water of the three campsites were culture-negative. A survey of maintenance practices by the RV users at the campsites revealed that chlorine disinfection of the water tanks was rarely performed. To prevent the possibility of *Legionella* infections, RV owners should implement regular chlorine disinfection of their water tanks and follow the recommended maintenance guidelines according to their owner's manuals.

## 1. Introduction

Organisms belonging to the genus *Legionella* have been clearly linked to contaminated potable and other water sources [1, 2]. Thermophilic properties possessed by these organisms often result in heavy colonization of heated water systems [3]. The organisms can reach high numbers in underchlorinated, heated, and agitated water. Recreational vehicle motor homes (RVs) have become very popular conveyances for travel in the United States during the past twenty-five years. A study conducted in 2011 by RV analyst Dr. Richard Curtin at the University of Michigan showed that the number of RV-owning households grew to 8.9 million households, up from 7.9 million in 2005 [4]. A popular destination for persons traveling by RV is to a sunny and warm climate where these vehicles may remain parked for extended periods of time. Ambient temperatures in resort areas may be expected to produce conditions within RV water storage tanks to easily support the growth of *Legionellae*, particularly if the tank is not properly disinfected on a regular basis. Moreover, luxuriant growth should be favored if the vehicle is parked in locations where it is exposed to hours of direct sunlight and the water is unused. We investigated RV water reservoirs as possible sources of *Legionella* infection in response to a case of fulminant pneumonia in a patient in which *Legionella pneumophila *was cultured from both the patient and the RV water reservoir.

## 2. Case Report

A 50-year-old previously healthy man who smoked occasionally and drank moderately attended an antique car exhibition in rural Pennsylvania in the fall of 1993 with several other enthusiast friends. The group traveled by RV and lived in the vehicle for several days using water for drinking and bathing directly from the storage tank. The water supply was replenished when required by refilling the tank with local tap water from a municipal system. 

Several days after returning from the event, the patient developed malaise and fatigue along with subjective fever and sweats. The patient was admitted to the hospital because of an abrupt onset of severe right flank pain. Initial suspicion of ureteral colic led to intravenous pyelography, which, while negative for ureterolithiasis, disclosed a dense right-lower-lobe pulmonary infiltrate. The patient was admitted and given i.v. ceftriaxone and erythromycin and nasal oxygen. Diffuse bilateral pulmonary infiltrates and respiratory insufficiency ensued. Attempts to maintain satisfactory oxygen saturation with nasal CPAP were unsuccessful and after several days of hospitalization, the patient was intubated and mechanically ventilated. 

Review of the original and several subsequent Gram-stained sputum samples showed many inflammatory cells, but bacteria were not evident. Wet mount, acid-fast-bacillus, and Wright stains of respiratory secretions were negative as was a direct fluorescent antibody stain for *Legionella pneumophila*. Cultures of these samples yielded no growth. The patient's condition failed to improve. On the sixth hospital day, fever became more pronounced and because of concern that the rise in temperature could be related to one of the antibiotics or *Candida* superinfection, erythromycin was discontinued and fluconazole was begun. Over the next two days the patient appeared moribund. At the suggestion of the infectious diseases consultant, erythromycin was reinstituted and methylprednisolone was begun in desperation. Within 48 hours the patient's oxygenation had improved. 

Additional cultures of respiratory secretions performed in the Savannah River Site research laboratory, Aiken, S.C., yielded *Legionella pneumophila*. Clinical resolution followed over the subsequent 10 days and the patient fully recovered. In an attempt to trace the patient's point source of exposure to the *Legionellae*, a water sample was obtained from the water reservoir of the RV in question. *Legionella pneumophila *was also isolated from the environmental sample.

## 3. The Study

### 3.1. Methods

Water samples were collected from faucets (*n* = 17) and or water tanks (*n* = 11) from 20 different RVs located at three different campsites in Petersburg, GA (*n* = 14), Ridge Road, GA (*n* = 4), and Modac, SC (*n* = 2). Control water samples (1 L at each location) for *Legionella* culturing were also obtained at a potable water tap at the three different campsite locations. Faucets and water tanks of the RVs were sampled by removing 1 L of water into a sterile screw cap bottle. If the water source had been previously chlorinated, 0.5 mL of 0.1 N sodium thiosulfate was added to the samples. Environmental samples were processed and cultured at the CDC according to CDC protocol “Procedures for the Recovery of *Legionella* from the Environment.” Direct fluorescent antibody staining was used for confirmation testing. A questionnaire regarding RV usage, cleaning and maintenance, and water treatment, as well as symptoms of recent respiratory illness was administered to the RV operators. 

### 3.2. Results

#### 3.2.1. Culture Results

Cultures from 4 of the 20 RVs (20%) yielded several different *Legionella* spp. (Table 1). Of the 28 water samples, 4 water faucets (24%) and one water tank (9%) were culture-positive for four *Legionella* spp: *L. pneumophila* (serogroups 1 and 6), *L. anisa*, *L. feeleii*, and *L. quateriensis*. Two of the four positive RVs contained *L. pneumophila*. 

#### 3.2.2. Demographics

All of the RVs in the study were registered in southern states including: TX, GA, FL, SC, VA, and NC except for one registered in the state of OH. The four RVs culture-positive for *Legionellae* were registered in different states, GA, SC, TX, and TN (Table 1). The average model year of all the RVs included in the study was 1997 and the average model year of all of the four positive RVs was also 1997. No specific make or model was associated with a positive *Legionella* culture. 

#### 3.2.3. Maintenance

Fifty percent of all the RVs surveyed had RV general maintenance performed within the last year with 60% of RVs having the water tank cleaned within the last year. Only one reported cleaning the water tank with chlorine. Others reported draining and or flushing as the cleaning procedure.

Forty-five percent of all the RVs would have the water tank drained when the RV was put in storage. However, only one of the RV owners treated the RV tank with chlorine before putting the water tank back into use. 

#### 3.2.4. Respiratory Health Survey

Two of the four RVs positive for *Legionella* spp. had respiratory complaints in the users (Table 1). An additional two RV occupants from the culture-negative RVs had respiratory complaints, including one RV user with a history of a dry cough treated with antibiotics (possible diagnosis of allergies) and a second RV user with a history of pneumonia treated with antibiotics (past medical history of allergies). 

## 4. Discussion

Legionellosis is an important concern for public health professionals and persons involved with maintaining building water systems. In a recent review of causes of outbreaks associated with drinking water in the U.S., summarizing the data from the Collaborative National Waterborne Disease and Outbreak Surveillance System, *Legionella* was responsible for 28.6% of the 84 drinking water outbreaks and 80.0% of drinking water-related deaths from 2001 to 2006 [5]. *Legionella* was the source of all of the acute respiratory illness outbreaks caused by drinking water from 1971 to 2006. Most of the legionellosis outbreaks were associated with *L. pneumophila* (70.8%) and most occurred in hospitals, health care facilities, or nursing homes. Community water systems were involved in 87.5% of the outbreaks and 12.5% in noncommunity water systems. Other outbreaks occurred in hotels, motels, lodges, and inns (16.7%) with additional outbreaks occurring in apartments and condominiums (12.5%). While *L. pneumophila* causes a majority of the reported cases of legionellosis in the U.S., two of the three additional *Legionella* spp. isolated in our study, *L. anisa* and *L. feeleii*, have also been associated with human disease [6].

Although approximately 11–32% of potable water supplies contain *Legionella* spp., our case of fulminant *Legionella* pneumonia acquired from an RV water tank and demonstration of *Legionella* in RV water tanks is unprecedented. The factors that lead to legionellosis from water sources are not always completely understood, but in this case we can postulate that an improperly sanitized RV water tank and warm weather conditions could allow for the multiplication of the *Legionellae. *The subsequent aerosolization of the bacteria during showers and bathing could be a risk factor for transmission of the pathogen.

Legionellosis is considered a preventable illness because controlling the growth of the *Legionella* in water reservoirs will prevent cases of the disease. Therefore, effective control and decontamination programs for the prevention of *Legionella* colonization should be instituted for portable water supplies such as RV water tanks. In surveying the RV users on their cleaning and maintenance of the water tanks, it was noted that chlorine disinfection was rarely performed. To safeguard the quality of tank water and prevent the possibility of *Legionella* infections, RV owners should implement regular chlorine disinfection of their water tanks and follow the recommended maintenance guidelines according to their owner's manuals.

## Figures and Tables

**Table 1:** *Legionella* spp. isolated, demographics, maintenance, and associated respiratory health issues of RVs positive for *Legionellae. *

RV no.	State	Specimen source	*Legionella* isolated	CFU/mL	General RV maintenance	Cleaning of tank within the last year	RV tank storage	Health problems in RV user
4	GA	Faucet	*L. feeleii *serogroup 1	0.15	No	No	Drain in winter	No
6	SC	Faucet	*L. pneumophila* serogroup 1	25	No	Yes	Drain and treat with chlorine	No
8	TX	Faucet	*L. quateriensis* *L. pneumophila* serogroup 6	2.6 6	Unknown	Yes	Always in use	Cough/allergies
17	TN	Water Tank Faucet	*L. anisa *(D-1497) *L. quateriensis* *L. anisa *(D-1497)	0.15–2.0 0.15–0.65 1.5–2.75	No	Yes	Always in use	Wife-history of bronchial pneumonia
